# A randomised controlled trial and cost-consequence analysis of traditional and digital foot orthoses supply chains in a National Health Service setting: application to feet at risk of diabetic plantar ulceration

**DOI:** 10.1186/s13047-018-0311-0

**Published:** 2019-01-08

**Authors:** D. J. Parker, G. H. Nuttall, N. Bray, T. Hugill, A. Martinez-Santos, R. T. Edwards, C. Nester

**Affiliations:** 1 School of Health Sciences, University of Salford, Salford, UK; 2 East Lancashire Hospitals NHS Trust, Burnley, UK; 3 Centre for Health Economics and Medicines Evaluation, Bangor University, Bangor, UK

**Keywords:** Foot orthotic, Biomechanics, Diabetes, Plantar pressure, Cost, Health economics, Supply chain

## Abstract

**Background:**

Diabetic foot ulceration is a considerable cost to the NHS and foot orthotic provision is a core strategy for the management of the people with diabetes and a moderate to high risk of foot ulceration. The traditional process to produce a custom-made foot orthotic device is to use manual casting of foot shape and physical moulding of orthoses materials. Parts of this process can be undertaken using digital tools rather than manual processes with potential advantages. The aim of this trial was to provide the first comparison of a traditional orthoses supply chain to a digital supply chain over a 6 month period. The trial used plantar pressure, health status, and health service time and cost data to compare the two supply chains.

**Methods:**

Fifty-seven participants with diabetes were randomly allocated to each supply chain. Plantar pressure data and health status (EQ5D, ICECAP) was assessed at point of supply and at six-months. The costs for orthoses and clinical services accessed by participants were assessed over the 6 months of the trial. Primary outcomes were: reduction in peak plantar pressure at the site of highest pressure, assessed for non-inferiority to current care. Secondary outcomes were: reduction in plantar pressure at foot regions identified as at risk (> 200 kPa), cost-consequence analysis (supply chain, clinician time, service use) and health status.

**Results:**

At point of supply pressure reduction for the digital supply chain was non-inferior to a predefined margin and superior (*p* < 0.1) to the traditional supply chain, but both supply chains were inferior to the margin after 6 months. Custom-made orthoses significantly reduced pressure for at risk regions compared to a flat control (traditional − 13.85%, digital − 20.52%). The digital supply chain was more expensive (+£13.17) and required more clinician time (+ 35 min). There were no significant differences in health status or service use between supply chains.

**Conclusions:**

Custom made foot orthoses reduce pressure as expected. Given some assumptions about the cost models we used, the supply chain process adopted to produce the orthoses seems to have marginal impact on overall costs and health status.

**Trial registration:**

Retrospectively registered on ISRCTN registry (ISRCTN10978940, 04/11/2015).

**Electronic supplementary material:**

The online version of this article (10.1186/s13047-018-0311-0) contains supplementary material, which is available to authorized users.

## Background

Diabetic foot ulceration is a complex condition, which requires regular clinical assessment, and wound management to prevent deterioration or infection with considerable cost to the National Health Service (NHS). The cost of wound care alone can range from £2140 to £8800 per diabetic foot ulcer, with much greater cost if this leads to amputation [1]. Foot orthoses are recommended to reduce forefoot plantar pressures in people with diabetes [2] and reducing peak plantar pressure to below 200 kPa is demonstrated to reduce the risk of re-ulceration [3]. The supply chain of customised foot orthoses includes an initial clinical decision making process to evaluate risk and to inform the specification and design of a product, followed by manufacture within physical (fit to shoe and fit to foot) and time constraints. This is driven by foot parameters (e.g. foot shape) and clinical information (e.g. risk status), but is also influenced by pragmatic issues such as material availability, cost and procurement constraints [4] and footwear choices made by patients [5]. These factors are known to influence the effects of foot orthoses and thus each part of the supply chain may impact on foot orthoses efficacy.

In a traditional or manual, supply chain foot shape is captured using plaster of Paris or foam impression boxes [4]. However, in a digital supply chain the foot surface is digitally scanned, a processe that is more repeatable but produces different orthoses geometries than the manual techniques [6]. Owings et al. [7] integrated plantar pressure data with foot shape in a digital orthoses supply chain to produce superior forefoot off-loading. Other work used computational models to optimise pressure relief [8]. These digital approaches are impossible to implement using traditional supply chain processes, which rely on manual identification of anatomical features (e.g. metatarsal heads). It follows that if supply chains use different data and processes to inform orthoses design, then pressure relief might also vary.

There are potential process benefits of using digital rather than traditional supply chains. Orthoses designs and processes are quantified, easier to control, adjust, and repeat, and data and information are permanently recorded and portable. Digital processes allow for standardised templates and design steps which can reduce the number of manual tasks and could be time saving if automated. There is also less physical waste and reduced space requirements (plaster, impression boxes, and storage of these). These potential advantages are only valuable, however, if the efficacy of the orthoses is not inferior to that of orthoses made through traditional manual processes. Whilst reduction in plantar pressure is the primary objective in using off-loading foot orthoses, this is assumed to be a pre cursor to reduced risk of ulceration and thereafter improvement in quality life [9]. Few studies have included these measures in their evaluation of foot orthoses designs and supply, though their inclusion has been advocated [9, 10].

Digital processes also involve a different financial model, with need for equipment, software, space, and staff time and training at both clinical and production sites. Any benefits accrued, whether in orthosis efficacy or supply process, must justify any additional costs. Few studies have evaluated cost issues and none have considered these data alongside plantar pressure and quality of life data to evaluate the difference between traditional and digital foot orthoses supply chains. Only Paton [10] measured plantar pressure changes, quality of life and some aspects of supply chain economics, but this compared prefabricated to custom made foot orthoses for people with diabetes. Comparisons of digital and traditional foot orthoses supply processes have focused on the resultant orthoses geometry and immediate pressure relief, [3, 8, 11–13], with longitudinal studies limited to comparisons between traditional and sham or prefabricated orthoses [9, 10, 14].

The aim of this study was to compare, over 6 months, plantar pressure, health-related quality of life and health service use in patients receiving orthoses through a digital supply chain compared to a traditional orthotic supply chain.

## Methods

### Trial design

This was a pragmatic parallel group randomised controlled trial with repeated measures assessing non-inferiority and cost-consequence analysis of traditional and digital foot orthoses production methods. Testing was conducted at the Royal Blackburn Hospital (East Lancashire NHS Trust) subsequent to ethical approval from institutional (HSCR15–89) and health service committees (REC ref.: 15/YH/0392) (registered trial, ISRCTN10978940).

### Outcomes

The primary outcome was the percentage reduction in peak plantar pressure at the site of highest forefoot plantar pressure. It was not ethical to provide a control orthotic on a longitudinal basis due to the high risk of ulceration in the cohort and as such a non-inferiority analysis was conducted. Secondary outcomes were the number of regions of interest (ROI) where plantar pressure was > 200 kPa; the percentage peak pressure reduction for all ROI; self-rated health-related quality of life and capability at 6 months; and service use costs at 6 months.

### Non inferiority margin

The use of the non-inferiority approach outlined here is in accordance with the FDA guidance for industry [15]. A non-inferiority margin (NIM) of 11.29% was established as a fixed margin in advance of data collection. The NIM was based on a pooled data analysis of prior studies which compared foot orthoses to flat control insoles [11, 13, 16], with an orthotic effect on peak pressure of 14.5% (95% Confidence Interval 11.29–17.77%). To ensure that the NIM represented the entire effect of orthoses in terms of pressure reduction the lower bound of the Confidence Interval (CI) was used, without the application of a clinically acceptable level of difference [see Additional file 1].

### Sample size

A priori power analysis for a non-inferiority test was based on data from previous work (*n* = 41) demonstrating an effect size of − 11.9% (standard deviation of difference = 15.2%) [17]. A sample size of 22 participants per arm was calculated (0.05 significance level, 80% power). Sample size was calculated using online software [18].

### Randomisation

Randomisation sequence was generated using a randomization plan generated using online software [19], in which 60 subjects were randomised into 10 blocks. This sequence was generated by a researcher independent of the trial team and a series of sealed envelopes were produced.

### Data collection

**Fig. 1:**
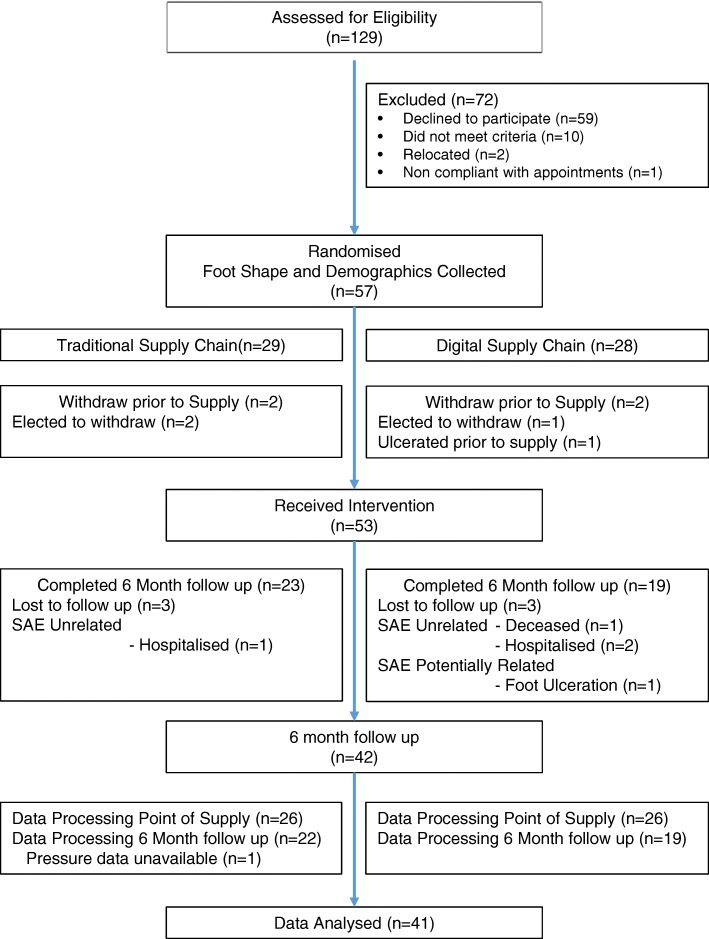
Consort Diagram for progression of participants through the study

Records of existing service users were screened to identify patients with diabetes, peripheral neuropathy and a moderate/high risk of ulceration. Potential participants were invited to a screening appointment (Fig. 1).

The study comprised three visits:

(1) potential participants were screened against inclusion/exclusion criteria (Table 1) by an orthotist and thereafter provided informed consent. Demographic data were collected and foot risk status determined as either moderate (one of; loss of sensation, peripheral vascular disease or signs of callus or deformity) or high (previous ulceration or amputation or more than one moderate risk factor present) for each foot using the SCI-DC framework [20] (Table 2). A foot related medical history was recorded (e.g prior ulceration, minor surgery, other interventions).

**Table 1:** Screening Criteria

Inclusion Criteria	Exclusion Criteria
Aged between 40 and 85 years	Have had prior foot or ankle surgery
Have Diabetes diagnosed by a medical practitioner	Have had major injury to the lower limb (e.g. fracture)
Have modular or bespoke footwear ^a^	Have had prior or active chronic foot or leg ulceration within last 2 years
Have all normal foot structures present	Require heel pressure reduction intervention
Be able to walk without a stick for 100 m	Have had prescription foot orthoses via the department in the last 12 months
Have sensory neuropathy ^b^	Absence of foot pulses assessed via Doppler or palpation
Capable of providing informed consent to participate	Comorbidities (Ischaemia, Renal, Charcot Arthropathy

^a^ provided by the orthotics department, ^b^ assessed by 10 mg monofilament and vibratory perception at less than 3 out of 10 sites on the foot and ankle

**Table 2:** Point of supply and Demographics

		Traditional Supply Chain			Digital Supply Chain		
Patient Demographics	*n*	26			26		
Age	(Years)	61.4	±	10.0	66.3	±	10.5
BMI	(kg/m^3^)	31.3	±	8.6	31.1	±	5.0
Gender	(M/F)	23	/	3	22	/	4
Clinical Factors							
History of Smoking	(No/Yes)	17	/	9	16	/	10
Previous Ulceration	(No/Yes)	12	/	14	17	/	9
Palpable foot pulses^a^	(No/Yes)	24	/	2	22	/	4
Foot Risk Status	(Moderate/High)	12	/	14	14	/	12
Neuropathic Symptoms Score	(0–4)	2.3	±	1.4	2.7	±	1.3
Footwear and Orthotics							
Duration in Orthotics Service	(Years)	6	±	4.5	6.4	±	4.9
Footwear Used	(Stock/Modular/Bespoke)	5 / 19 / 2			1 / 23 / 2		

^a^Pulses were detectable by Doppler in all cases

(2) At a second visit the appropriate orthoses were fitted by an orthotist. Plantar pressure data was collected (Pedar insoles, Novel, Germany) whilst participants walked in their orthotic insoles and a control insole (flat 3 mm Poron). The order of testing was randomised. Walking speed was established using timing gates (Brower Timing Systems, Draper, Utah, USA) prior to data collection and maintained within +/− 5% [21]. A minimum of three 10 m walks were recorded in each of the two conditions, yielding approximately 30 steps per foot after excluding periods of acceleration/deceleration.

The EQ-5D-5 L and the ICECAP-A were used to assess health-related quality of life (HRQoL) [22] and capability (well-being beyond health) [23] respectively. The EQ-5D is a generic, validated HRQoL measure [22]. The EQ-5D-5 L was analysed to produce an index score between 0 (state of death) and 1 (perfect health). The ICECAP-A is a validated capability measure focussing on well-being beyond health and is scored from 0 (no capability) to 1 (full capability) [23].

Participants took the orthoses home with instructions for use daily in everyday footwear. Adherence to use of orthoses was monitored by monthly telephone follow-up to ensure regular use with the intention to exclude participant data from subsequent analysis where there is an apparent substantial lack of use of the orthosis.

(3) At a 6 month visit plantar pressure, EQ-5D-5 L, ICECAP-A and NHS service use was measured. NHS service use was examined using a client service receipt inventory (CSRI) and participants were asked to recall the last 6 month use of NHS services relating to foot conditions [see Additional file 2]. To investigate economic differences in the two supply chains, clinical process times (excluding research activities) were documented and compared as part of a cost analysis.

### Interventions

Orthotics in both supply chains were modifications of a foot size specific template. The template included: medium density EVA rearfoot (30–40 ShoreA) with minimum 6 mm thickness under the heel; a medial arch profile and heel cup (10 mm). The forefoot (area distal to the end of the medial arch) was a minimum of 6 mm Poron (20 ShoreA). Modifications to relieve pressure (cavities, material substitutions or additions) were made on a patient by patient basis to reflect real practice (Table 3). All orthoses were finished with a leather top cover.

**Table 3:** Modifications to Orthotics

Type of Orthotic Modification	Traditional Supply Chain	Digital Supply Chain
Design Modification		
Local removal of material	1	1
Local softening of material	2	3
Addition of metatarsal pad or bar		2
Addition of Wedge or Skive		2
Maintenance and Repair		
Repair or Glue of top cover	14	9
Replacement of top cover	1	
Insole Damaged/Replaced	1	

Design modifications were made on a patient by patient basis to relieve pressure. Maintenance and repair was made when required to reflect normal practice within the Orthotics service

For the traditional supply chain a foam impression box was used to capture foot shape at visit 1 and a written prescription form completed. Devices were manufactured by filling the impression box with plaster, heat moulding material to the cast, and hand finishing. Manufacture was external to the orthotics department and blind to the research study (Beagles Orthopaedic, UK).

For the digital supply chain foot shape was captured using a weight bearing foot scan at visit 1 (Inescop, Spain). Static plantar pressure distribution data was recorded during barefoot standing (F-Mat, Tekscan, USA). These data were integrated in CAD software (iCAD Pan, Inescop, Spain) to allow adjustment of the orthotic template (from Salford Healthcare Ltd) by the orthotist on a patient by patient basis. Static pressure data was used to adjust the position of modifications (Table 3). Digital models were used for CNC milling (Victor 1200, UK) and orthoses finished by hand.

To allow for non-inferiority assessment a flat 3 mm Poron insert very similar to that used in studies which established efficacy of orthoses was used as a control.

### Data processing

#### Plantar pressure data

Data for the foot which had the highest clinical risk status based on SCI-DC framework [20] was selected for analysis, when both feet had equal risk status the left foot data was used. Plantar pressure data was segmented with Matlab (MatWorks, Inc. Version 9.0, USA) into hallux, 1st metatarsal head, and metatarsal heads 2–5, consistent with [3]. Peak pressure was calculated for each region in each step and the mean calculated by averaging across all footsteps.

For each participant the site of highest peak pressure in the control insole (flat Poron insole) was identified and compared to peak pressure at the same site when wearing the orthotic to determine an orthotic effect (% increase or decrease in peak pressure). For each participant all regions which had a mean peak pressure > 200 kPa in the control insole were designated as regions of interest (ROI) [3], with each participant having 0–3 ROIs. For each ROI the orthotic effect on peak pressure was determined.

#### Health service use costs

Service use was costed using published national unit costs available at the time of data collection [24, 25]. Staff costs were calculated using NHS Band 6 point 25 and Band 5 point 19, inflated by on-costs, overheads and capital overheads, as recommended by Curtis et al. [24]. Cost of the orthoses were £60 for traditional and £50 for digital supply chains.

#### Health status data

The EQ-5D produces an index score between 0 (state of death) and 1 (perfect health). At the time of analysis, a validated UK value set was not available to score the EQ-5D-5 L, and therefore, a crosswalk value set was used to calculate utility values based on the EQ-5D-3 L scoring system [26]. ICECAP-A was used to produce a total capability score from 0 (no capability) to 1 (full capability) [23].

### Statistical analysis

#### Plantar pressure data

##### Primary analysis

Plantar pressure data was assessed per-protocol (completers) using SPSS (IBM SPSS Statistics: Version 24, USA). This approach ensured that comparisons made between groups at set time points were equivalent in terms of regular orthoses use. This further ensured that factors such as material performance, known to impact on biomechanical performance [27] and compliance did not bias the results. Post screening 57 participants were randomised to the digital (*n* = 28) or traditional (*n* = 29) supply chains, allowing for some expected drop out in each group. At fitting appointment 53 participants remained in the study (26 digital, 27 traditional supply chain) (Fig. 1). At the 6 month visit 42 participants (19 digital, 23 traditional supply chain) had used the devices regularly, 11 had withdrawn (5 due to adverse events (4 digital, 1 traditional) and 6 lost to follow up (3 digital, 3 traditional) (Fig. 1). Peak plantar pressure data in the control and orthotic insoles was not normally distributed.

##### Secondary analysis

Within supply chain analysis was conducted to compare control to orthotic using Wilkoxon signed ranks test. Between supply chains group variance was assessed using the Mann-Whitney U test, comparing control-to-control and orthotic-to-orthotic. Orthotic effect was calculated as the percentage change in peak pressure from control to orthotic insoles, this data was normally distributed and was compared between supply chains using independent t-tests.

For each supply chain, the orthotic effect was assessed for non-inferiority against a pre-established NIM [28]. To establish non-inferiority the lower bound of the two-sided CI for the orthotic effect was compared to the NIM (11.29%). If the lower bound of CI is greater than the NIM then the orthotic effect is not inferior to the established effect of orthoses based on the literature.

#### Health economic data and health status outcomes

A cost-consequence analysis was undertaken and service use was compared between the two supply chains. Mean differences in costs between the two interventions were calculated using nonparametric bootstrapping, and run on 5000 iterations, to produce 95% confidence intervals around these differences [29]. The analysis was undertaken from an NHS perspective. NICE recommend an NHS and personal and social services perspective, as the societal perspective risks bias against individuals who are retired or unable to work. The NHS perspective focuses on costs and benefits directly associated with health care treatment, disease management and associated administrative costs [30]. Discounting was not undertaken as the length of interventions did not exceed 12 months. Independent samples t-tests were used to assess differences in mean change (point of supply-6 month) in HRQoL and wellbeing between the two trial arms.

## Results

Over the 6 months six participants were lost to follow up (3 in each arm), there were 4 unrelated adverse events (1 traditional, 3 digital) and one foot ulceration at a ROI (digital arm) a further 1 dataset was excluded from pressure data analysis due to corrupted files Fig. 1. The groups were comparable in terms of clinical and risk status at the start of the trial (Table 2) and there was no statistically significant difference between peak pressures for the control condition for either site of highest pressure or ROI analysis (Tables 4 and 5), suggesting a comparable pressure profile across both groups. After 6 months a significantly increased peak pressure at the site of highest pressure for the digital group was found in both control and orthotic conditions compared to the traditional Group (*p* < 0.01), suggesting a change to the pressure profile.

**Table 4:** Pressures at ROIs

	*n*	Control			Orthotic				Orthotic Effect					
		Mean (SD)			Mean (SD)				Mean (SD)		95% CI		Wilcoxon Rank	
		(kPa)			(kPa)				(%)		(LB - UB)		Z	*p*
Traditional Supply Chain														
0 M	42	307.92 (84.87)			263.56 (98.19)			↓	13.85 (20.24)		(7.55–20.16)		−3.395	0.001
6 M	25	276.53 (53.78)			240.96 (69.94)			↓	11.94 (24.11)		(1.98–21.89)		−2.704	0.007
Digital Supply Chain														
0 M	42	308.88 (88.93)			244.08 (87.83)			↓	20.52 (15.79)		(15.54–25.50)¥		−5.208	0.000
6 M	32	298.95 (84.57)			271.75 (80.49)			↓	8.01 (18.69)		(1.27–14.75)		−2.431	0.015
Difference		T-D	M-W U	*p*	T-D	M-W U	*p*		T-D	t	*p*	95% CI		
0 M		−0.96	880	0.986	19.48	1001	0.287		−6.67	1.675^*^	0.098	(−1.26 to 14.59)		
6 M		−22.42	343	0.359	−30.79	297	0.098		3.93	0.693^**^	0.491	(−15.28 to 7.43)		

*LB* Lower Bound, ↑: Increase in pressure for custom orthotics compared to flat control, ↓: Decrease in pressure for custom orthotics compared to flat control. Statistical significant difference between control and orthotic was assessed via related samples Wilcoxon Rank test ǂ: *p* < 0.05, ф: *p* < 0.01. T-D: Mean for Traditional group minus Mean for Digital Group. Non-Inferiority was assessed against a predefined margin of 11.29%. ¥:Lower bound of the 95% Confidence interval for the intervention effect was greater than the NI margin

**Table 5:** Site of Highest Pressure

	*n*	Control			Orthotic				Orthotic Effect					
		Mean (SD)			Mean (SD)				Mean (SD)			95% CI	Wilcoxon Rank	
		(kPa)			(kPa)				(%)			(LB - UB)	Z	*p*
Traditional Supply Chain														
0 M	26	309.67 (101.33)			261.52 (110.67)			↓	14.91 (20.56)			(5.89–22.87)	−2.679	0.007
6 M	22	251.12 (64.98)			209.84 (63.83)			↓	16.71 (17.25)			(9.06–24.35)	−3.652	0.000
Digital Supply Chain														
0 M	26	318.95 (109.78)			240.03 (113.36)			↓	24.43 (20.18)			(16.27–32.58)¥	−4.026	0.000
6 M	19	321.32 (90.33)			279.74 (90.05)			↓	12.41 (20.23)			(2.65–22.16)	−2.334	0.020
Difference		T-D	M-W U	*p*	T-D	M-W U	*p*		T-D	t	*p*	95% CI		
0 M		−9.28	355	0.756	21.49	279	0.280		−9.520	−1.695**	0.096	(−20.80 to 1.76)		
6 M		−70.20	316	0.005	−69.90	312	0.007		4.300	0.726**	0.473	(−7.72 to 16.31)		

*LB* Lower Bound, ↑: Increase in pressure for custom orthotics compared to flat control, ↓: Decrease in pressure for custom orthotics compared to flat control. Statistical significant difference between control and orthotic was assessed via related samples Wilcoxon Rank test ǂ: *p* < 0.05, ф: *p* < 0.01. T-D: Mean for Traditional group minus Mean for Digital Group. Non-Inferiority was assessed against a predefined margin of 11.29%. ¥:Lower bound of the 95% Confidence interval for the intervention effect was greater than the NI margin

### Non-inferiority

**Fig. 2:**
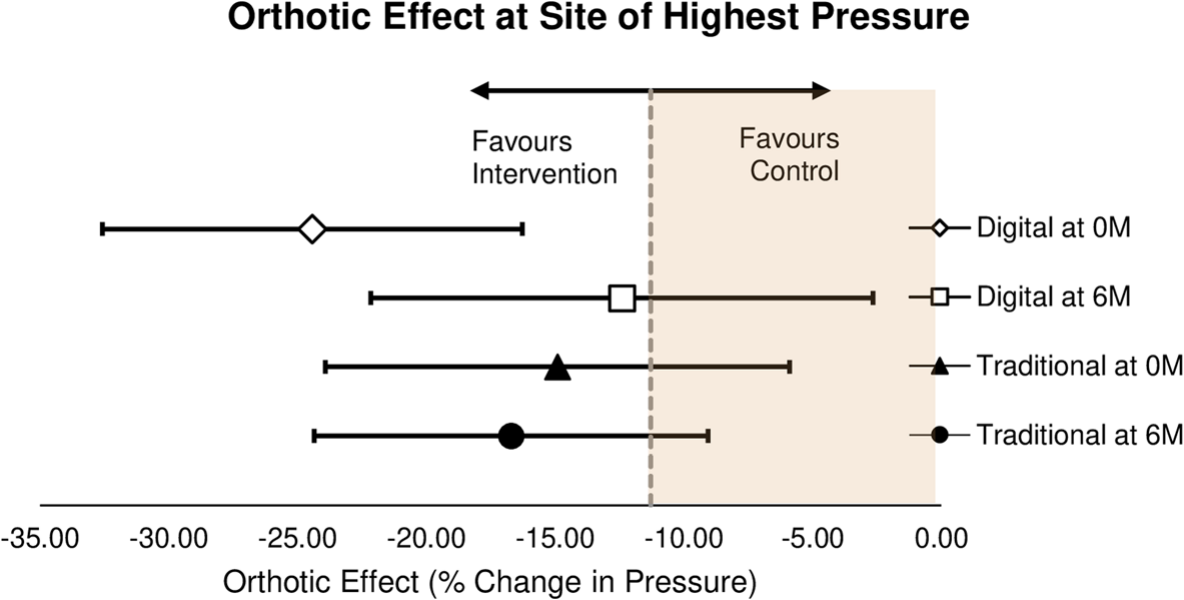
Non-inferiority assessment of Orthotic Effect at Site of Highest Pressure. Error bars indicate 2-sided 95% CIs. The dashed line at x = − 11.29 indicates the non-inferiority margin (NIM). The yellow tinted region to the right of x = − 11.29 indicates the zone of inferiority. Digital at 0 M lies wholly left of zero indicating a reduction in pressure compared to control and wholly to the left of the NIM indicating that this is non-inferior. Digital at 6 M, Traditional at 0 M and Traditional at 6 M all lie left of zero indicating a reduction in pressure with orthotics but the lower boundary of their confidence intervals are to the right of the NIM meaning non-inferiority is not demonstrated

The orthotic effect in the traditional supply chain at the site of highest pressure and all ROI was inferior to the NIM at point of supply (Tables 4 and 5). The orthotic effect of the digital supply chain was non-inferior to the NIM at point of supply and was superior (*p* < 0.1) to the traditional supply chain. However, at 6 months, the orthotic effect of both the traditional and digital supply chains was inferior to the NIM (Fig. 2).

### Site of highest peak plantar pressure

**Fig. 3:**
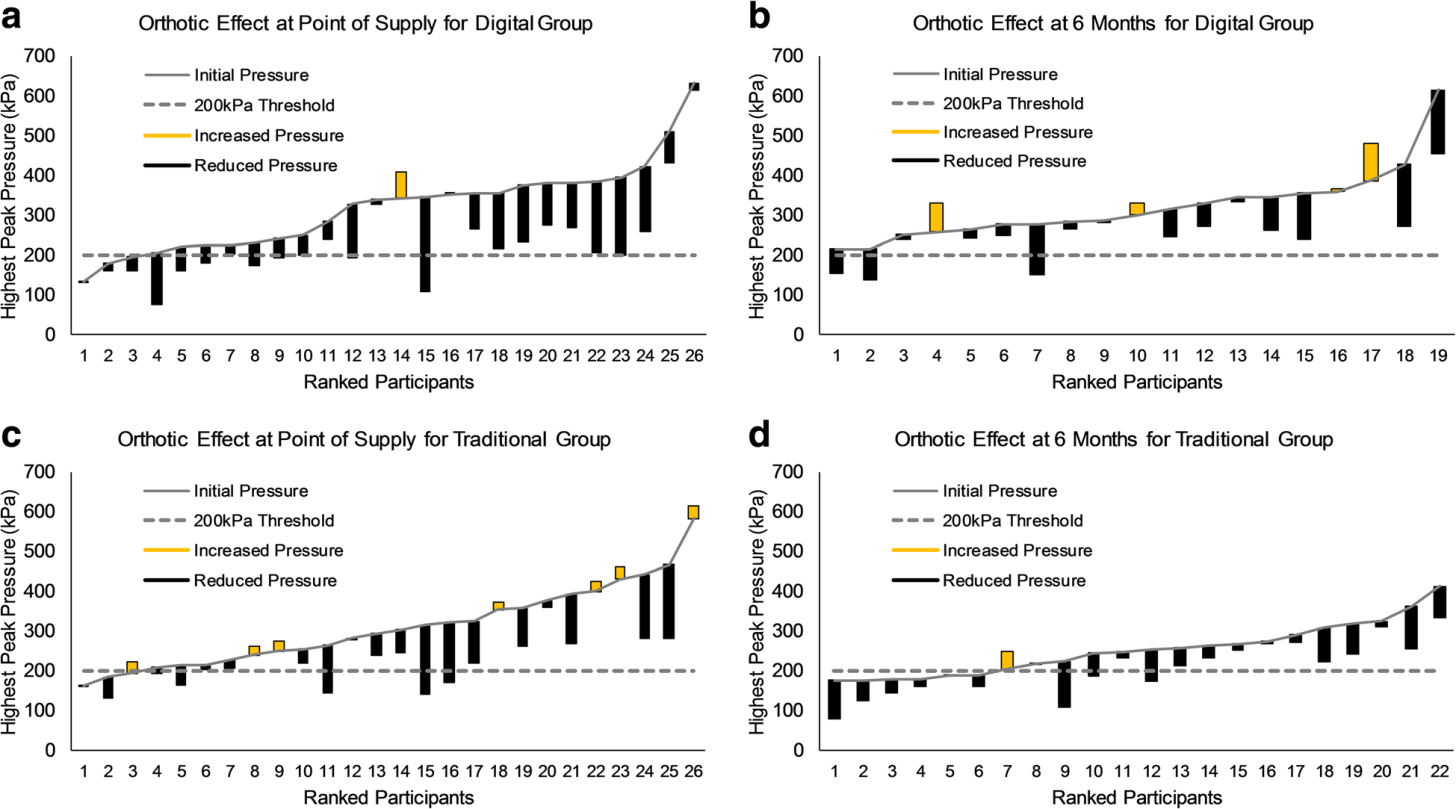
Orthotic effect at Site of Highest Pressure within the forefoot. Data for orthotic effect on peak pressure data at site of highest pressure in each particiant, ordered based on pressure measured without orthotic (control condition). Initial peak pressure recorded without orthotics (**a**) Digital supply chain at point of supply, (**b**) Digital Supply Chain at 6 Months, (**c**) Traditional supply chain at point of supply, (**d**) Traditional Supply Chain at 6 Months

At the point of supply and at 6 months the effect of orthoses on peak pressure was statistically significant (*p* < 0.05), demonstrating a reduction of pressure with the orthotic insoles in both supply chains vs control insoles (Table 4). At point of supply, peak pressure at the site of highest pressure was reduced below 200 kPa in 17 and 39% of participants in the traditional and digital groups respectively (Fig. 3). This orthotic effect was not statistically significantly different between supply chains at the point of supply or 6 months.

### Regions of interest

**Fig. 4:**
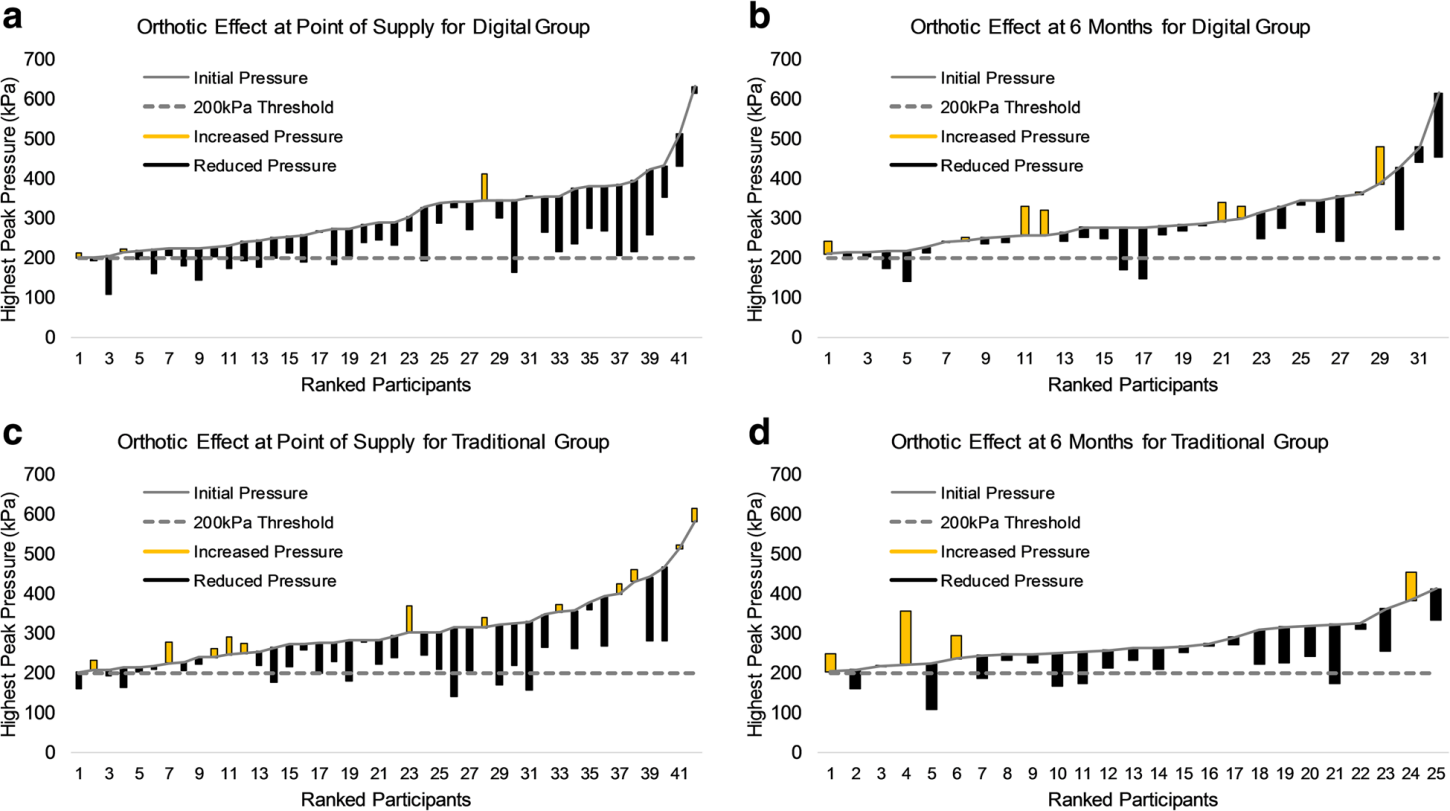
Orthotic effect on Region of Interests (Peak Pressure > 200 kPa) within the forefoot. Data for orthotic effect on peak pressure from all regions in which peak pressure was over 200 kPa in control condition, ordered based on pressure measured without orthotic (control condition) . Initial peak pressure recorded without orthotics (**a**) Digital supply chain at point of supply, (**b**) Digital Supply Chain at 6 Months, (**c**) Traditional supply chain at point of supply, (**d**) Traditional Supply Chain at 6 Months

For both the traditional and digital groups over 50% of regions assessed were considered to be high risk (> 200 kPa). The use of orthoses significantly reduced peak pressure by > 10% at point of supply and > 8% at 6 months for ROIs in both the traditional and digital supply chains (p < 0.05) (Table 5). At point of supply, orthoses reduced the number of ROIs by 21% for the traditional group and 33% for the digital group. After 6 months, taking into account participant dropout, orthoses reduced the number of ROIs by 24% for the traditional group and 16% for the digital group (Fig. 4).

### HRQoL

Participants in the traditional supply chain showed slightly better overall HRQoL and self-rated health status effects compared to the digital supply chain at 6 month follow-up, but vice versa for capability (Table 6). The changes were not statistically significantly different between the supply chains.

**Table 6:** HRQoL and wellbeing: Effectiveness results and statistical significance for quality of life measures

	Traditional Supply Chain^a^			Digital Supply Chain^b^			Digital v Traditional	
	Point of supply (SD)	6 month (SD)	Mean change (SD)	Point of supply (SD)	6 month (SD)	Mean change (SD)	Mean effect at 6 months	Independent samples *t* test
EQ-5D	0.645 (0.728)	0.685 (0.261)	0.040 (0.190)	0.728 (0.239)	0.671 (0.227)	−0.057 (0.191)	−0.097	t(40) = 1.644, *p* = 0.108
EQ-VAS	63.91 (23.97)	62.65 (21.13)	−1.26 (19.78)	71.05 (18.83)	65.26 (19.33)	−5.79 (21.62)	−4.529	t(40) = 0.708, *p* = 0.483
ICECAP-A	0.730 (0.181)	0.693 (0.195)	−0.037 (0.105)	0.801 (0.173)	0.829 (0.132)	0.028 (0.107)	0.064	t(38) = −1.908, *p* = 0.064

^a^*N* = 23 for EQ-5D and EQ-VAS, 22 for ICECAP-A, ^b^*N* = 19 for EQ-5D and EQ-VAS, 18 for ICECAP-A. Due to a lack of statistical significance there is no indication that either intervention was effective at improving HRQoL, health status or capability in this study

### Cost analysis

The traditional supply chain cost an average of £72.63 per participant and the digital £85.68 (difference of £13.17, Table 7). The digital supply chain took over three times longer (53:10 min [23:08] versus 17:39 [11:04] respectively) and led to differences in staff time costs (£12.63 for traditional versus £35.68 for digital), partly offset by the higher orthotic cost in the traditional supply chain (£60 versus £50 in the digital).

**Table 7:** Staff time and costs for the two supply chains

Staff activity	Mean staff time^a^ (SD)	Mean staff Cost	Mean total cost^b^
Traditional supply chain (*n* = 17)			
Clinical Assessment^c^	05:18 (01:46)	£3.79	
Foot Shape Capture^c^	00:56 (00:40)	£0.67	
Written Prescription^c^	01:49 (01:38)	£1.31	
Medical Notes^c^	09:32 (09:43)	£6.82	
Modify Impression Box^c^	00:04 (00:15)	£0.04	
Total	17:39 (11:04)	£12.63	£72.63
Digital supply chain (*n* = 18)			
Clinical Assessment^c^	06:43 (01:56)	£4.81	
CAD Design^c^	17:07 (11:09)	£12.25	
Medical Notes^c^	11:37 (11:21)	£8.31	
Foot Shape Capture^d^	06:17 (04:56)	£4.49	
Configure Scans^d^	05:17 (03:33)	£3.78	
Align Scans^d^	06:10 (03:42)	£4.41	
Total	53:10 (23:08)	£35.68	£85.68

^a^time format minutes:seconds (mm:ss), ^b^including orthotic cost of £60 for TSC and £50 for DSC, ^c^ Activity completed by NHS Orthotist, ^d^ Activity completed by NHS Technician

### Service use

On average participants in the traditional supply chain accessed an additional £194 worth of NHS services in the 6 month follow-up period (Table 8) compared to those in the digital supply chain, though this was not statistically significant. Differences in inpatient service use explain the variance. Two participants’ accessed inpatient services, both in the traditional supply chain arm, and incurred high costs (£2116.04 each). Removing inpatient service use reduces the overall mean difference in service use costs to £17.79, remaining higher in the traditional supply chain arm.

**Table 8:** NHS service use costs relating to foot conditions or diabetes over 6 month follow-up period

Service use	Traditional Supply Chain (*n* = 24)			Digital Supply Chain (*n* = 20)				Mean difference (95% CI^a^)
	3 month (SD)	6 month (SD)	Total (SD)	3 month (SD)	6 month (SD)	Total (SD)		
PRIMARY CARE AND COMMUNITY SERVICES								
General Practitioner	£22.46 (52.55)	£28.58 (53.42)	£51.04 (89.63)	£4.90 (15.08)	£30.63 (67.86)	£35.53 (70.86)	↓	-£15.52
Practice Nurse	£5.58 (10.41)	£6.75 (13.67)	£12.33 (16.79)	£3.91 (9.77)	£5.58 (14.26)	£9.49 (15.91)	↓	-£2.84
District Nurse	£0.93 (4.56)	£0.00	£0.93 (4.56)	£0.00	£0.00	£0.00	↓	-£0.93
Diabetes Specialist Nurse	£0.00	£3.39 (12.36)	£3.39 (12.36)	£4.38 (10.94)	£6.25 (12.66)	£10.63 (15.72)		£7.24
Chiropodist	£9.00 (15.60)	£13.13 (20.65)	£22.13 (26.02)	£7.50 (12.14)	£11.40 (9.67)	£18.90 (15.33)	↓	-£3.23
Dietician	£0.79 (3.88)	£0.00	£0.79 (3.88)	£1.43 (6.37)	£1.90 (6.20)	£3.33 (8.56)		£2.53
Other	£0.00	£2.05 (7.09)	£2.05 (7.09)	£0.49 (2.20)	£0.00	£0.49 (2.20)	↓	-£1.56
Total	£38.76 (58.87)	£53.89 (65.72)	£92.65 (99.49)	£22.60 (27.39)	£55.76 (81.55)	£78.36 (86.74)	↓	-£14.29 (−67.28 to 22.19)
SECONDARY CARE: OUTPATIENT								
Orthotics Department	£8.92 (30.21)	£17.83 (40.73)	£26.75 (27.33)	£5.35 (23.93)	£5.35 (23.93)	£10.70 (32.93)	↓	-£16.05
Chiropodist	£1.50 (7.35)	£0.00	£1.50 (7.35)	£0.00	£0.00	£0.00	↓	-£1.50
Dietician	£0.00	£0.00	£0.00	£0.95 (4.25)	£0.95 (4.25)	£1.90 (8.50)		£1.90
Dietetics Department	£0.00	£0.00	£0.00	£3.45 (15.43)	£0.00	£3.45 (15.43)		£3.45
Diabetes Consultant	£0.00	£0.00	£0.00	£12.60 (56.35)	£0.00	£12.60 (56.35)		£12.60
Other	£1.50 (7.35)	£6.00 (13.71)	£7.50 (18.32)	£3.60 (11.08)	£0.00	£3.60 (11.08)	↓	-£3.90
Total	£11.92 (30.99)	£23.83 (44.26)	£35.75 (55.76)	£25.95 (86.04)	£6.30 (24.08)	£32.25 (91.60)	↓	-£3.50 (−35.35 to 29.21)
SECONDARY CARE: INPATIENT								
Chiropody Unit	£0.00	£88.17 (431.93)	£88.17	£0.00	£0.00	£0.00	↓	-£88.17
Other	£0.00	£88.17 (431.93)	£88.17	£0.00	£0.00	£0.00	↓	-£88.17
Total	£0.00	£176.34 (597.42)	£176.34 (597.42)	£0.00	£0.00	£0.00	↓	-£176.34 (− 364.84 to 0)
SERVICE TOTAL	£50.68 (71.34)	£254.06 (602.06)	£304.74 (592.87)	£48.55 (103.56)	£62.06 (82.85)	£110.61 (145.90)	↓	-£194.13 (− 393.52 to 0.35)

^a^ 95% bootstrapped confidence interval, based on 5000 replications ↓ demarks a reduction in cost for Digital Supply Chain compared to Traditional Supply Chain

## Discussion

The two supply chains provided orthoses that were largely comparable in terms reductions in plantar pressure after a period of continued use despite significant differences at point of supply. Since HRQoL, capability and economic data also failed to reveal any difference between supply chains, any benefits of one process over the other must lie outside of the parameters we quantified. It follows that pressure relief is not an adequate basis for choosing one supply chain over the other.

This outcome is sensitive to the assumptions in our research design. It has been noted that preference-based measures of HRQoL can be insensitive for patients with impaired mobility [31–33]. For instance, the EQ-5D-5 L offers five relatively narrow levels of mobility: no problems, slight, moderate, severe or extreme problems walking. For many orthoses users these options lack nuance, and therefore may be too blunt to fully capture changes in HRQoL related to orthotic interventions. Assumptions may also have an impact on the use of cost data. A recent survey identified that digital and traditional supply chains are in use internal and external to the NHS, which will distribute costs differently than our models [4]. Also, an unexpected outcome was that digital supply chain assessments took longer than in the traditional supply chain. This was likely because the digital processes were new to staff and could be streamlined with practice. This illustrates that possible savings might not be immediate as there would be a learning period and longer term cost analysis is required. Indeed financial modelling might need to cover several years in the case of purchasing capital equipment (for both supply chains). The difference between chains of £13.17 might also be impacted if other suppliers are used compared to those used in the study.

The issue of cost and seeking economies in processes might drive supply chain innovation. Some parts of the processes, such as CNC manufacture, are naturally designed for large volume and potentially continuous operation. Capitalising on the potential this offers requires other parts of the supply process to be adapted, such as ensuring sufficient volumes of orthoses are required and facilities management outside of normal working hours. These issues could be addressed by distributing different parts of the supply chain across a network of contractors to seek economies associated with scale, or using a single supply chain to support multiple health organisations. The context for costs might also be very sensitive to local arrangements, such as availability of extra space without capital expenditure, and in situ staff capacity and skills. One of the motivations behind this trial was the potential to ‘future proof’ foot orthoses supply by moving to a digital context and later exploring innovations such as additive manufacturing. Moving to a digital context requires a much more thorough analysis of service, supply chain models, innovation opportunities and longer term economic planning than our trial allowed.

The orthoses used were typical of those recently reported [4] and the levels of pressure relief were similar to those reported by Burns [9] (~ 18% using Pedar) but less than those reported by Paton [10] (~ 30% using Tekscan). These differences may be due to variability between different measurement systems used [34] and the use of a ‘no insole’ control instead of sham orthotic by Paton. Changes in efficacy were observed between point of supply and 6 months suggesting insole integrity or durability may have been affected, this is demonstrated by Paton 2014 who investigated effect of wear on insole performance [27]. Results confirm that foot orthoses made bespoke for each patient can move patients from a classification of “at risk of ulceration” (> 200 kPa) to lower risk category [3]. This suggests the orthoses tested were largely typical of those in the literature and fit for purpose. That orthoses produced by different supply chains and across different published studies both demonstrate pressure relief, might suggest that pressure reliving effects at the level of groups of patients are not sensitive to the nuances of different orthoses design. This is somewhat contrary to the within patient differences that we and others have reported when small orthoses design features are manipulated, such as changes in material and geometry [8, 9]. This suggests that there is not consistent “best” approach overall, but that a number of approaches can deliver the intended pressure relief for an individual patient.

Several limitations are important to note. Differences in plantar pressure when wearing the control insole at the point of supply and 6 months suggests a change to the pressure profile. This may relate to changes in the participants foot structure or tissues associated with disease progression and the risk status [35], though this was not recorded. The effect size used to power this study was a conservative estimate and was in line with the non-inferiority margin identified. However, the sample size calculations were powered by effect sizes for plantar pressure outcomes, as this was assumed a prerequisite for other outcomes being pertinent. There is therefore insufficient statistical power to make definitive assessments for cost effectiveness of the supply chains. If the digital supply chain were to be adopted as routine practice a number of additional economic factors would need to be considered, including the initial cost of software, equipment and space, and the ongoing need to train staff. More detailed process cost modelling is required to better understand the impact of these costs in specific service context. The non-inferiority margin used for this study was based on a limited number of studies; more robust studies to assess orthotic effect are required to ensure a more robust and clinically meaningful boundary can be established. The follow-up period was 6 months and practitioners replace orthoses at different intervals, so a longer term follow up is relevant [4]. We did not control footwear beyond it being suitable to accommodate orthoses. Control of footwear has been recommended for assessment of orthotic effect [35], however this was a pragmatic trial including an evaluation of the supply chains in their entirety requiring the use of real practice footwear selected by orthotists and patients.

## Conclusion

Custom made foot orthoses produced by both traditional and digital supply chains provide significant reduction in pressure compared to a flat 3 mm Poron insert and retain this functionality throughout 6 months of regular use. There is only a marginal difference in terms of cost and health status between supply chain processes used to design, produce and maintain foot orthoses. Custom made orthoses were also demonstrated to reduce the number of regions of the foot identified as at risk due to high pressures. Effectiveness was found to be higher in the digital supply chain however, there was no statistically significant difference between supply chains after 6 months of use. Further to this after 6 months, orthoses were found to be inferior to a pre-defined margin based on prior studies demonstrating effective pressure reduction. Further studies to assess the long-term effectiveness of foot orthoses are needed to ensure appropriate design, production and monitoring can be implemented for management of risk with foot orthoses in the diabetic foot.

## Additional files

Additional file 1: This file outlines the process for estimation of the Non-Inferiority Margin based on previous data. (DOCX 24 kb)

Additional file 2: Client Service Receipt Inventory, this document demonstrates the format for collection of health service use data within this study. (PDF 394 kb)

## Abbreviations

CI: Confidence interval
CSRI: Client service receipt inventory
HRQoL: Health-related quality of life
NHS: National health service
NIM: Non-inferiority margin
ROI: Region of interest
SD: Standard deviation
