# Supplementary material for: A randomised controlled trial and cost-consequence analysis of traditional and digital foot orthoses supply chains in a National Health Service setting: application to feet at risk of diabetic plantar ulceration
Source: J Foot Ankle Res. 2019 Jan 8;12:2. doi: 10.1186/s13047-018-0311-0 (PMC6325812; doi:10.1186/s13047-018-0311-0)
Supplement: Supplementary file 1 — This file outlines the process for estimation of the Non-Inferiority Margin based on previous data. (DOCX 24 kb) [file 13047_2018_311_MOESM1_ESM.docx]

**Additional File 1: Non-Inferiority Margin**

Non-Inferiority analysis is used to determine that the intervention being studied is atleast as effective as the current standard of practice [1,2]. To do this a non-inferiority margin (NIM) must be defined based on literature evidence, this can also include clinical judgement. The NIM is recommended by the Food and Drug Administration to be the lower bound of the 95% confidence interval around the treatment effect [2].

Calculation of Non-Inferiority Margin for pressure reduction with custom foot orthoses

Data was identified from literature which demonstrated superiority of the handmade orthotic design methodology over a placebo or sham insole [3–5]. Papers were identified to align to the methods of the proposed study with a similar clinical population.

To establish a suitable non-inferiority margin the following steps were taken.

1. For each source the following steps were taken to establish Mean and Standard Deviation (SD) of control (flat) and intervention (custom orthotic) conditions. Data are presented in table A1.
   1. Use reported Mean and Standard Deviation
   2. When no absolute values were reported but data was presented in the format of a graph with Mean and SD bar values were estimated using Image J software [6] to measure the height of bar. The y axis was used as calibration scale.

**Table A1. Data from the literature for control (Flat Insert) vs intervention (Custom Orthotic)**

|  | **Study A*** | | **Study B** | | **Study C** | |
| --- | --- | --- | --- | --- | --- | --- |
|  | n=6 | | n=21 | | n=11 | |
|  | Control | Intervention | Control | Intervention | Control | Intervention |
| Mean | 215 | 173 | 302 | 255 | 281 | 252 |
| SD | 71 | 52 | 109 | 81 | 112 | 68 |
| *No Mean or SD values presented data calculated from graph. | | | | | | |

1. The Mean and Standard Deviation were combined via weighted average methods. Results presented in Table A2.
   1. Weighted Average ($x_{g}$) of the Mean data.
      $\bar{x_{g}}=\frac{\sum_{i=1}^{g} (n_{i}\bar{x}_{i})}{\sum_{i=1}^{g} n_{i}}$
   2. Weighted Average of the Standard Deviation ($\sigma_{g}$) data.

$$\sigma_{g}=\sqrt{\frac{1}{n_{g}-1}\cdot(\sum_{i=1}^{g} {n-1}_{i}\sigma_{i}^{2}+\sum_{i=1}^{g} n_{i}{(\bar{x}_{i}-\bar{x_{g}})}^{2})}$$

In which; $\bar{x_{g}}$ is the weighted average of the grouped data,$\bar{x_{i}}$ is the mean for each study, $n_{g}$ is the total subject number of the grouped data, $n_{i}$ is the subject number for each study, $\sigma_{g}$ is the weighted standard deviation of the grouped data, $\sigma_{i}$ is the Standard deviation for each study.

1. Mean Percentage change and Standard Deviation were calculated for weighted control vs intervention data. Results presented in Table A2.

- 1. Mean Percentage Change
  $z=\frac{100(x-y)}{x}$
  2. Standard Deviation of the Percentage Change

$$\sigma_{z}=z(\frac{\sigma_{x}}{x}+\frac{\sigma_{y}}{y})$$

In which; $z$ is the percentage change, $x$ is mean of control, $y$ is mean of intervention, $\sigma_{z}$ is the standard deviation of the percentage change, $\sigma_{x}$ is the standard deviation of the control, $\sigma_{y}$ is the standard deviation of the intervention.

**Table A2. Weighted average data for control (Flat Insert) vs intervention (Custom Orthotic)**

|  | **Control** | **Intervention** |  | **% Change** |
| --- | --- | --- | --- | --- |
| Mean | 282.18 | 241.18 |  | 14.53% |
| SD | 107.00 | 77.84 |  | 10.20% |

1. 95% Confidence intervals were determined for the Percentage Change
   1. Margin of Error

$$Margin of Error= Z_{a/2}\cdot\frac{\sigma_{z}}{\sqrt{n_{g}}}$$

In which; $Z_{a/2}$ is the confidence coefficient, *a* is the confidence level, $\sigma_{z}$ is the standard deviation of the percentage change, *n* is the sample size of the pooled data.

The following values were applied; a = 0.95, $\sigma_{z}$ = 10.20, n = 38

The corresponding Z value for a=0.95 is 1.96, therefore:

**Margin of Error** = 1.96*(10.20/√38) = **3.24**

- 1. The lower and upper Confidence Intervals were determined.

**Lower Bound of 95% Confidence Interval is 11.29% reduction in kPa**

Upper Bound of 95% Confidence Interval is 17.77% reduction in kPa

1. Non inferiority Margin Defined

Non inferiority requires test data to demonstrate a lower bound of the 95% Confidence interval for the **percentage reduction in pressure as greater than 11.29%**

References:

[1] Mauri L, D’Agostino RB. Challenges in the Design and Interpretation of Noninferiority Trials. N Engl J Med 2017;377:1357–67. doi:10.1056/NEJMra1510063.

[2] FDA, CDER, CBER. Non-Inferiority Clinical Trials to Establish Effectiveness Guidance for Industry 2016. https://www.fda.gov/downloads/Drugs/Guidances/UCM202140.pdf (accessed July 3, 2018).

[3] Ashry HR, Lavery LA, Murdoch DP, Frolich M, Lavery DC. Effectiveness of diabetic insoles to reduce foot pressures. J Foot Ankle Surg 1997;36:268–71. doi:10.1016/S1067-2516(97)80071-3.

[4] San Tsung BY, Zhang M, Arthur Fuk Tat Mak, Margaret Wan Nar Wong. Effectiveness of insoles on plantar pressure redistribution. J Rehabil Res Dev 2004;41:767–74.

[5] Bus SA, Ulbrecht JS, Cavanagh PR. Pressure relief and load redistribution by custom-made insoles in diabetic patients with neuropathy and foot deformity. Clin Biomech 2004;19:629–38. doi:10.1016/j.clinbiomech.2004.02.010.

[6] Schneider C, Rasband W, methods KE-N, 2012 undefined. NIH Image to ImageJ: 25 years of image analysis. NatureCom n.d.
