# Supplementary material for: A randomised controlled trial and cost-consequence analysis of traditional and digital foot orthoses supply chains in a National Health Service setting: application to feet at risk of diabetic plantar ulceration
Source: J Foot Ankle Res. 2019 Jan 8;12:2. doi: 10.1186/s13047-018-0311-0 (PMC6325812; doi:10.1186/s13047-018-0311-0)
Supplement: Supplementary file 2 — Client Service Receipt Inventory, this document demonstrates the format for collection of health service use data within this study. (PDF 394 kb) [file 13047_2018_311_MOESM2_ESM.pdf]

Telephone Review 1 - Service Use Receipt

Interviewer instructions: **Please complete all sections** of the table to show the services that the participant has used **since the screening appointment**.

Patient Identity Number: | 0 | 0 | 0 | 0 | 0 | 0 | 0 | 0 | 0 | 1 |

Date of Visit: ______________________

1 Community Based Service Use

Service [Used by participant]	No. of home visits			No. of visits to surgery or clinic			Provider agency (please cross)				Average duration of contact (minutes)	Cause/ Reason for Visit
	Foot	Diabetes	Other	Foot	Diabetes	Other	NHS	Local authority	Voluntary org	Private org		
General practitioner (GP)												
Practice nurse (GP Clinic)												
District Nurse												
Diabetes Specialist Nurse (DSN)												
Chiropodist												
Dietician												
Health Visitor												
Other												

2 Out-Patient Care Service Use

Service [Used by participant]	No. of visits to hospital			Provider agency (please cross)				Average duration of contact (minutes)	Cause/ Reason for Visit
	Foot	Diabetes	Other	NHS	Local authority	Voluntary org	Private org		
Orthotics Department									
Chiropodist									
Dietician									
Dietetics									
Consultant									
Other									

3 In-Patient Care Service Use

Service [Used by participant]	No. of visits to hospital			Provider agency (please cross)				Average duration of contact (minutes)	Cause/ Reason for Visit
	Foot	Diabetes	Other	NHS	Local authority	Voluntary org	Private org		
Chiropodist									
Dietician									
Dietetics									
Consultant									
Other									
